# Efficiency of Double Layered Microencapsulated Probiotic to Modulate ProInflammatory Molecular Markers for the Management of Alcoholic Liver Disease

**DOI:** 10.1155/2014/715130

**Published:** 2014-05-22

**Authors:** Sumeha Arora, Indu Pal Kaur, Kanwaljit Chopra, Praveen Rishi

**Affiliations:** ^1^Department of Microbiology, Panjab University, Basic Medical Sciences Building, BMS Block Panjab University, Chandigarh 160014, India; ^2^University Institute of Pharmaceutical Sciences, Panjab University, Chandigarh 160014, India

## Abstract

Alcohol-related disorders are one of the challenging current health problems with medical, social, and economic consequences. Endotoxemia, oxidative stress, and release of a variety of inflammatory molecules are established mediators in alcoholic liver injury (ALD). Probiotics like *L. plantarum* though were reported to attenuate ALD, their *in vivo* health benefits are limited by their survival and sustenance in the adverse gut conditions. Therefore, to enhance their *in vivo* performance, chitosan coated alginate beads entrapping *L. plantarum* were prepared, characterized, and evaluated for their efficacy against ALD in rats. Following chronic alcohol exposure, rats developed endotoxemia, showed enhanced levels of liver enzyme markers, NF-*κ*B levels, and increased cytokines such as TNF-**α** and IL12/p40 subunit, and reflected significant histological changes in the intestine and liver. However, cosupplementation with double layered microencapsulated probiotic significantly (*P* < 0.05) reduced the levels of endotoxemia, serum transaminases, NF-*κ*B, and cytokines complemented with restoration of normal histoarchitecture of the intestine and liver. It is being documented here for the first time that the probiotics have the potential to inhibit IL-12/p40 subunit which is a recently explored potential marker for developing novel therapeutic agents. This study reveals that microencapsulation of probiotics may offer a biopharmacological basis for effective management of ALD.

## 1. Introduction

Alcohol-related disorders (ALD) are one of the challenging current health problems with far reaching medical, social, and economic consequences [1–3]. Several lines of investigations [4, 5] indicate that alcohol abuse induces endotoxemia, activation of transcription factor NF-*κ*B, and release of a variety of inflammatory mediators including TNF-*α*, IL-1*β*, and IL-6 responsible for mounting oxidative stress culminating into liver injury.

Use of probiotics to manage the alcohol-induced endotoxin mediated liver injury is attributed to a variety of its health benefits including immunomodulatory and anti-inflammatory effects. A randomized trial carried out by Kirpich et al. in 2008 and 2012 showed an improvement of liver function tests and restoration of normal bowel flora upon administration of probiotics for the treatment of alcoholic liver disease. Further to this,* Lactobacillus* GG also ameliorates oxidative stress and intestinal permeability in alcoholic liver injury. Probiotics such as* Lactobacillus plantarum*,* Lactobacillus* GG, and* Bifidobacterium bifidum* have demonstrated a significant reduction of oxidative stress and restoration of normal bowel flora [6–11].

Several important mechanisms by which the probiotic provides various health benefits include modification of the gut microbiota, competitive adherence to the mucosa and epithelium, strengthening of the gut epithelial barrier, and modulation of the immune system of the host. Evidence also demonstrates that probiotics communicate with the host by pattern recognition receptors, such as toll-like receptors which modulate key signaling pathways, such as nuclear factor-*κ*B which either enhances or suppresses the downstream pathways [12].

However, major concern for the use of probiotics* in vivo* is that they must survive and sustain transit through the extreme conditions of the gut in large quantities to facilitate their colonization in the host to confer these benefits. In this context, encapsulation techniques may ensure greater survival of probiotic bacteria under gastric conditions. This is a method by which bacteria are protected from detrimental factors of environments such as high acidity (low pH), bile salts, molecular oxygen in case of obligatory anaerobic microbes, bacteriophages, and chemical as well as antimicrobial agents [13–15]. However, to the best of our knowledge, improved efficacy, if any, of microencapsulated probiotic against liver damage has not been assessed so far.

Amongst the encapsulation devices, microencapsulation in calcium alginate microparticles has been widely used owing to its ease of handling, nontoxic nature, and low cost.* Alginate* is a linear heteropolysaccharide extracted from algae, with two structural units consisting of D-mannuronic and L-guluronic acids and it forms hydrogels in the presence of divalent ions such as Ca^2+^. However, certain disadvantages are attributed to alginate beads including the susceptibility to acidic environments and deterioration of beads when subjected to monovalent ions or chelating agents which absorb calcium ions. These limitations can be efficiently compensated by coating alginate with suitable compounds so as to impart it with mechanical strength.* Chitosan* is a linear polysaccharide with negative charge arising from its amine groups which are obtained by deacetylation of chitin [16]. Chitosan has been used for coating the alginate capsules to provide strength and for continuous sustainable release of bacteria. Another advantage of chitosan, which adds to its use as a coating material, is its mucoadhesive properties, which prolongs the residence time of dosage allowing a sustained drug release at a given target site. Furthermore, mucoadhesive polymers can guarantee an intimate contact with the absorption membrane, providing the basis for a high concentration gradient as a driving force for passive drug uptake [17]. Anticipating the added advantage of encapsulated probiotics, in the present study, efficacy of these probiotic bacteria microencapsulated in a double layer of polymers was evaluated for the management of ALD.

## 2. Materials and Methods

### 2.1. Agents

Absolute ethanol (99.9%) was purchased from Brampton, Ontario. The probiotic microorganism* Lactobacillus plantarum* (MTCC 2621), used as a probiotic, was acquired from Microbial Type Culture Collection (MTCC), Institute of Microbial Technology, Chandigarh (India).

*L. plantarum* was cultivated in* Lactobacillus* MRS broth (1% inoculum) at 37°C for 18 hours. The cells were harvested by centrifuging the culture at 8000 rpm for 15 min at room temperature. The cells were washed twice with 0.1% peptone water and were suspended in 1 mL of the same.

### 2.2. Microencapsulation of Cells

*L. plantarum* was encapsulated in calcium alginate by the method of Krasaekoopt et al. 2003, 2004 [18, 19] via extrusion technique. Briefly, the suspended cells were dispersed in prepared 1%, 2%, and 3% sterile sodium alginate and kept for overnight stirring. The dispersion of* L. plantarum* in a solution of sodium alginate was then dropped into 1% sterile calcium chloride with stirring on a magnetic stirrer. The beads were left for hardening by continuous stirring for 2 hours. The formed* L. plantarum* encapsulated alginate beads (AL beads) were then coated with medium molecular weight chitosan (Sigma, India) using the method of Krasaekoopt et al. 2003 [18], in which the beads were suspended in chitosan solution (0.4 g of chitosan in 90 mL distilled water acidified with 0.4 mL of glacial acetic acid to achieve a final concentration of 0.4% w/v; pH 5.7–6) and were stirred for 1 hour. The beads were then filtered using Whatman filter paper 1 and were freeze dried (−60°C) under vacuum for storage (*n* = 6).* The alginate loaded microparticles were named as AL and AL beads coated with chitosan were referred to as AL-CA.*

#### 2.2.1. Characterization and Evaluation of AL and AL-CA Beads

*(1) Determination of Particle Size*. The particle size of the formed beads (AL and AL-CA beads) was determined in triplicate and mean size was recorded using particle size analyzer (Malvern instruments limited, Malvern, UK). 

*(2) Scanning Electron Microscopy (SEM) for Surface Morphology*. Freeze dried calcium alginate beads (AL and AL-CA beads) were coated with gold film under vacuum to modify the conducting materials and surface morphology was studied. 

*(3) Drug Entrapment Efficiency (DEE) of Probiotic Beads*. For bacterial enumeration, the beads (AL and AL-CA beads) were crushed in 1% sodium citrate solution while stirring for 60 minutes. Serial dilutions were made in 0.1% peptone water and spread plate on MRS Agar and incubated for 48 hours at 37°C (*n* = 6) and number of colonies forming units (cfu) was counted. Thereafter percentage of entrapment was calculated using the following formula:

(1) DEE% =log⁡cfu/100 g  of  prepared  beads×100log⁡cfu/mL  initially  loaded  in  the  alginate  mix.

(DEE = drug entrapment efficiency; cfu = colony forming units). 

*(4) Viability of Bacteria Postentrapment*. To assess the viability of entrapped bacteria the beads (AL and AL-CA beads) were crushed in 1% sodium citrate solution while stirring for 60 minutes. Serial dilutions were made in 0.1% peptone water and 0.1 mL was spread plated on MRS Agar plates and incubated for 48 hours at 37°C.

*(5) Determination of Porosity*. Beads (AL and AL-CA beads) were filled in a 10 mL graduated measuring cylinder up to the mark. The cylinder was tapped 500 times and the final volume was noted. Initial volume was kept the same in all the cases and the final volume gave the tap volume. The porosity was calculated according to the following equation and mean % porosity and standard deviation were recorded [16]:

(2) $$\begin{array}{c} \text{Porosity}=\frac{V_{b}-V}{V_{p}}\times 100 \end{array}$$

*V*_*b*_: bulk volume of the beads = 10 mL; *V*_*p*_: true/tap volume of the beads;

(3) $$\begin{array}{c} V=V_{b}-V_{p}. \end{array}$$

*(6) Viability of Entrapped Probiotic Bacteria in Simulated Gastric Fluid (SGF) and Sequentially in Simulated Intestinal Fluid (SIF).* Free probiotic cells and the beads (AL and AL-CA beads) were incubated in SGF without pepsin (dissolved 2.0 g of sodium chloride in 7.0 mL of hydrochloric acid and sufficient water to make 1000 mL, pH 1.2) for 4 hours and the samples were then sequentially transferred to SIF without pancreatin (dissolved 6.8 g of monobasic potassium phosphate in 250 mL of water, mixed, and added 77 mL of 0.2 N sodium hydroxide and 500 mL of water and adjusted the resulting solution to a pH of 6.8 ± 0.1, diluted with water to 1000 mL) for 2 hours and viable count was studied as described earlier [14]. 

*(7) In Vitro Release Studies*. Release study of probiotic beads (AL and AL-CA beads) was carried out in SIF under aseptic conditions. 100 mg beads were incubated at 37°C in test tubes containing 10 mL of SIF for 3 hours. At intervals of 1 hour, the supernatant from each tube was analyzed for cell count. For bacterial enumeration, serial dilutions of the supernatant were made in 0.1% peptone water and spread plate on MRS agar and incubated for 48 hours at 37°C. 

*(8) Bile Salt Tolerance*. The viability in the presence of bile salts was assessed by suspending the free cells and beads (AL and AL-CA beads) in MRS broth supplemented with 0.3% (w/v) bile salts (sodium deoxycholate and sodium taurocholate) for 4 hours and bacterial count was estimated as described earlier.

### 2.3. *In Vivo* Studies

#### 2.3.1. Ethics Statement

The experiment protocols were approved by the Institutional Animal Ethics Committee (approval ID: IAEC/282/dated 30/8/2012) and performed in accordance with the guidelines of Committee for the Purpose of Control and Supervision of Experiments on Animals (CPCSEA), Government of India, on animal experimentation. All efforts were made to minimize the suffering of animals.

#### 2.3.2. Animals

Female Wistar rats (200–250 g) were procured from Central Animal House, Panjab University, Panjab University, Chandigarh (India). The animals were housed under standard laboratory conditions, maintained on a 12 : 12 h light : dark cycle, and had* ad libitum* access to food (Ashirwad Industries, Pvt, Ltd., Punjab, India) and water. It has been reported in the literature that females develop more severe liver injury than males due to smaller amount of body water and lower activity of alcohol dehydrogenase enzyme (ADH) in stomach [20].

#### 2.3.3. Dosing

*Alcohol Dosing*. Rats were administered 10 g/kg of body weight/day of 35% (v/v) ethanol by oral gavage in double distilled water for two weeks. Thereafter, the dose was increased to 14 g/kg of body weight/day and was continued for 10 weeks through oral gavage [21].

*Probiotic Dosing*. 10^10^ cfu/mL of* Lactobacillus plantarum* was dispersed in 1 mL of PBS (pH 7.2). Chitosan coated alginate beads containing equivalent* L. plantarum* were dispersed in 1% carboxymethyl cellulose and were administered to rats through oral gavage.

#### 2.3.4. Experimental Design (Figure 3)

After an acclimatizing period, rats were randomly divided into the following four groups each comprising 10–12 rats as depicted in Figure 3. The dose of the alcohol group was selected on the basis of the previous study [21]. At the end of the experimental period (after 12 weeks), the rats were sacrificed by cervical dislocation. Livers were removed quickly, rinsed in cold phosphate buffer saline (0.05 M, pH 7.4), and stored at −62°C.

#### 2.3.5. Measurement of Blood Alcohol

After 10 weeks of alcohol administration, blood was taken from the tail vein 1.5 h and 2.5 h after gavage. Blood alcohol levels (BAL) were measured using the alcohol dehydrogenase kit from Sigma Chemical Co., USA.

#### 2.3.6. Plasma Endotoxin Assay

Endotoxin level in the plasma samples was measured using Toxin Sensor Chromogenic LAL Endotoxin Assay Kit (Hycult Biotech). Briefly, 50 *μ*L of plasma was incubated with 50 *μ*L of limulus amebocyte lysate (LAL) at 37°C for 45 min. After several subsequent reactions, the samples were read spectrophotometrically at 405 nm. The plasma endotoxin levels were calculated against a standard curve of endotoxin concentrations of 0.1, 0.04, 0.02, 0.01, and 0.005 EU/mL.

#### 2.3.7. Markers of Liver Damage

*Assessment of Liver Function*. Alanine aminotransferase (ALT) and aspartate aminotransferase (AST) enzyme activities in serum were determined using ERBA test kits (ERBA Diagnostics, Mannheim, Germany). Alkaline phosphatase (ALP) was estimated using Enzopak Diagnostic kit (Reckon Diagnostics, India).

*Histological Studies*. Liver and intestine tissues removed aseptically from the animals were cut into small pieces and fixed in 10% buffered formalin. Samples were dehydrated in different grades of alcohol, washed with xylene, and embedded in paraffin wax, and the sections were stained with hematoxylin-eosin and examined under the light microscope. Histological interpretation was done by Dr. B. N. Datta, Ex-Professor of Pathology, Post Graduate Institute of Medical Education and Research, Chandigarh (India).

*Mechanistic Studies*. Livers removed aseptically from the rats were rinsed in 0.05 M phosphate buffer saline (pH 7.4) (PBS). A 25% (w/v) tissue homogenate was prepared in PBS using a Potter Elvehjen homogenizer. 

*Assay for NF-κ* *B p50 Subunit*. Assay for NF-*κ*B/p50 subunit in the nuclear extracts was performed in all the groups by commercially available Transcription Factor Assay kit (Upstate Biotechnology, NY, USA) according to the manufacturer's instructions. This assay combines the principle of the electrophoretic mobility shift assay (EMSA) with the 96-well based enzyme linked immunosorbent assay (ELISA). Briefly, cellular extracts were prepared from liver tissue using Chemicon's Nuclear Extraction kit. During the assay, the capture probe, a double stranded biotinylated oligonucleotide containing the consensus sequence for NF-*κ*B, was mixed with the nuclear extract in the transcription factor assay buffer provided directly in the streptavidin coated plate and incubated for 2 h at room temperature. Plates were then washed to remove the unbound material. The bound NF-*κ*B transcription factor subunit p50 was detected with rabbit anti-NF-*κ*B p50 (specific primary antibody). HRP conjugated secondary antibody was then used for detection using 3,3,9,5,5,9-tetramethybenzidine (TMB/E) as the substrate and absorbance was read at 450 nm. Positive and negative controls were also run simultaneously [21]. 

*Assay for Tumor Necrosis Factor-*α* (TNF-*α*)*. Assay for TNF-*α* was performed by ELISA in the liver homogenates using commercially available cytokine assay kit (R&D Systems, USA) according to the manufacturer's instructions described by us earlier [21]. Briefly, standards and tissue homogenates were dispensed in the 96-well microtiter plates precoated with monoclonal antibody specific for rat TNF-*α*. To each of the designated wells, 50 *μ*L of assay diluent was added; the plates were sealed with acetate plate sealer and incubated at room temperature for 2 h. Plates were then washed five times with the wash buffer and 100 *μ*L of rat TNF-*α* conjugate was dispensed into each well. Plates were again sealed and incubated at room temperature for 2 h, after which they were washed five times with the wash buffer and 100 *μ*L of substrate solution was dispensed into each well. Plates were finally incubated at room temperature (in dark) for 30 min. 100 *μ*L of the stop solution was added into each well to stop the reaction and absorbance was read at 450 nm. The results were expressed as picogram/mL of the TNF-*α* released. The ELISA was sensitive to 5 picogram/mL of the TNF-*α* released. 

*Assay for IL12/p40 Subunit*. To check the levels of IL12/p40 subunit a double antibody sandwich ELISA was performed using the commercially available kit (Qayee-bio, China) according to the manufacturer's instructions. Briefly, standards (50 *μ*L) and test samples (10 *μ*L of liver homogenate + 40 *μ*L of sample diluent) were added to the 96-well microtiter plates precoated with monoclonal antibody specific for rat IL-12/p40 subunit. To these designated wells, 50 *μ*L of HRP labelled IL12/p40 subunit antibody was added and incubated for 60 minutes at 37°C. Following incubation, the excess liquid was discarded, dried, and washed five times with washing buffer. 50 *μ*L each of chromogens A and B was dispensed and incubated for 10 minutes at 37°C away from light. The assay was stopped by adding 100 *μ*L of stop solution and the absorbance was read at 450 nm.

## 3. Results

### 3.1. Characterization of Beads

#### 3.1.1. Size of Microparticles and Scanning Electron Microscopy (SEM) Images

**Figure 1:**
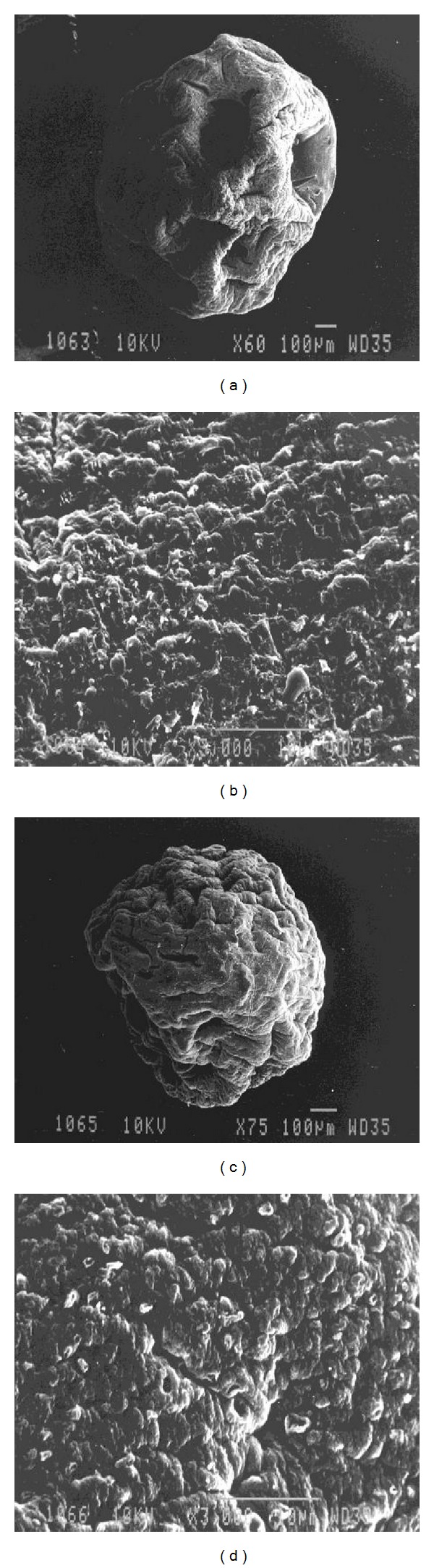
Scanning electron micrographs of (a) alginate beads containing probiotic (60x), (b) alginate beads containing probiotic (300x), (c) AL-CA beads containing probiotic (60x), and (d) AL-CA beads containing probiotic (3000x).

The alginate (AL) microparticles (Figure 1(a)) were spherical in shape with a wrinkled surface. The wrinkled surface can probably be due to the loss of water content during the lyophilization process. On the other hand, incorporation of medium molecular weight chitosan not only significantly (*P* < 0.05) increased the size of the probiotic loaded beads but also smoothened the surface of alginate microparticles (Figure 1(b)). The mean size of the AL beads was 69.2 ± 6.9 mm which was significantly (*P* < 0.05) lower than the AL-CA beads 80.4 ± 1.5 mm (Table 1).

#### 3.1.2. Entrapment Efficiency

Viable count determination of the used probiotic was repeated six times (*n* = 6) and the mean viable count was calculated. In all the cases, the initial cell count was kept in the range of 10.2–10.73 log cfu/mL. Maximum cell entrapping was observed to be 80% in the probiotic loaded beads where the concentration of sodium alginate was kept as 2% (Table 2). The viable cell count obtained was 8.15 ± 0.20 log cfu/100 mg in alginate beads. Further, on coating with chitosan, 77% entrapment efficiency was obtained.

#### 3.1.3. Porosity

The chitosan coated alginate beads entrapping probiotic (AL-CA) were more porous (95%) as compared to the alginate (AL) probiotic beads (84%).

#### 3.1.4. Viability of Entrapped Probiotic Bacteria in Simulated Gastric Fluid (SGF) and Sequentially in Simulated Intestinal Fluid (SIF)

To assess the likelihood of microencapsulated and free probiotic bacteria surviving passage through the stomach following oral administration, they were tested in simulated gastric fluid (SGF; pH-1.2) without pepsin for four hours and sequentially in simulated intestinal fluid (SIF; pH-7.4) without pancreatin for two hours.  Table 3 shows a significant decrease (*P* < 0.05) in cell count for free probiotic than AL beads and AL-CA beads. However, chitosan coated probiotic alginate beads (AL-CA) provided the best protection (71% of the bacteria survived after six hours in alkaline environment) than the AL (69.4% survival rate) and free probiotic (56.8% survival rate). The cell count of free probiotic gradually decreased by *≈*6 log units with incubation in SGF without pepsin for four hours. A decrease in cell count (*≈*4 log units) was also observed with AL beads. However, the viability of* Lactobacillus plantarum* was reduced by only *≈*2 log units when double encapsulation was provided with chitosan over alginate beads. In the alkaline environment, the cell viability of the free probiotic further was reduced to *≈*2 log units which shows that the free probiotic cannot tolerate the harsh environment of gastrointestinal pathway.

#### 3.1.5. *In Vitro* Release Study

**Figure 2:**
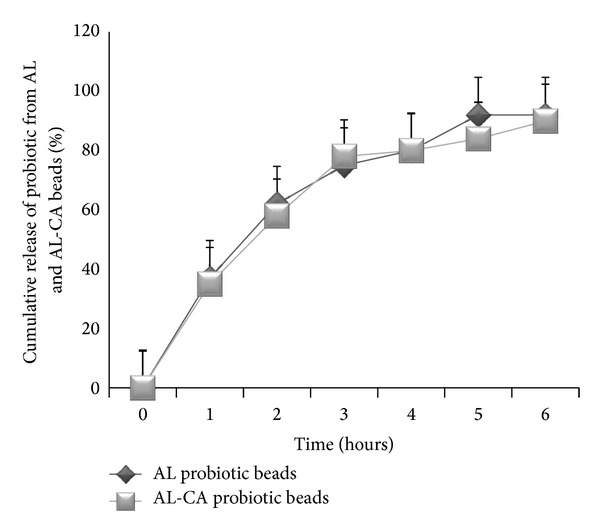
The* in vitro* release study for AL and AL-CA beads up till 6 hours. No significant difference was found for the two types of beads.

**Figure 3:**
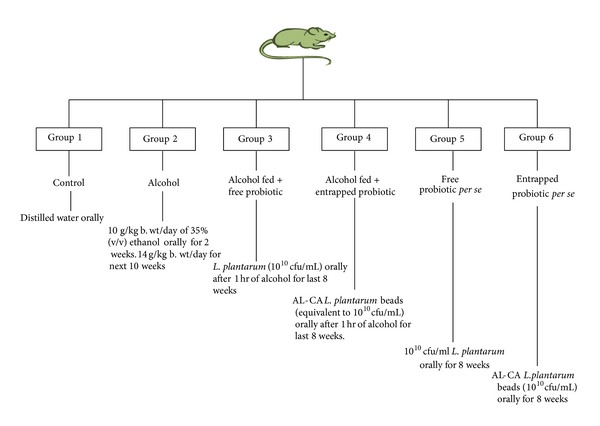
Diagrammatic representation of various treatment groups made for* in vivo* studies.

The* in vitro* release studies (Figure 2) showed that there was constant release of probiotic from both types of beads during the four-hour duration in the SIF. No significant difference (*P* < 0.001) was observed between the AL-CA and AL probiotic beads (~80%). The AL-CA beads could not affect the release of probiotic rather it offered better stability.

#### 3.1.6. Bile Salt Tolerance

Chitosan coated alginate microparticles encapsulating* L. plantarum* were the most effective (*P* < 0.05; 7.95 ± 0.87) in providing protection against bile salts (Table 4).

As the chitosan coated alginate beads (AL-CA) provided better protection in all the above-mentioned parameters to the probiotic than the alginate beads (AL), therefore, the former were used for further* in vivo* studies for chronic alcohol consumption.

### 3.2. *In Vivo* Studies

#### 3.2.1. Blood Alcohol Levels

After 12 weeks of regular alcohol administration, the blood alcohol levels were found to be significantly increased in the alcohol supplemented group when compared to other groups. Blood alcohol levels (BAL) 1.5 h and 2.5 h after ethanol administration by gavage in the alcohol group were 243.2 mg/dL and 198.6 mg/dL, respectively. BAL in the alcohol treated and chitosan coated probiotic alginate beads supplemented group were 214.7 and 176.1 mg/dL after 1.5 and 2.5 h of alcohol administration, respectively.

#### 3.2.2. Plasma Endotoxin Levels

**Figure 4:**
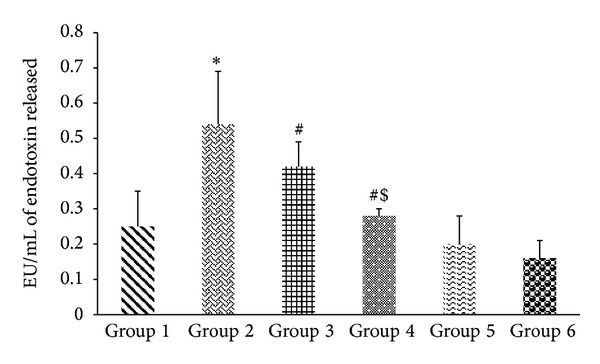
Effect of probiotic (free and encapsulated) on endotoxin levels in alcohol administered rats. Values are expressed as mean ± S.D. of eight different observations. *P < 0.05 versus group 1, group 5, and group 6; ^#^ *P* < 0.005 versus alcohol (Alc) (group 2); ^$^ *P* < 0.05 versus group 3.

The alcohol administered rats (group 2) suffered from significant endotoxemia (0.54 EU/mL) as compared to the control rats (0.16 EU/mL). The plasma endotoxin levels in alcohol administered and AL-CA supplemented group were significantly (*P* < 0.01) lower (0.312 EU/mL) than group 2 (Figure 4).

#### 3.2.3. Assessment of Liver Functions

Estimation of alanine, aspartate aminotransferases, and alkaline phosphatase is considered as biochemical markers for liver damage. Therefore, in the present study levels of ALT, AST, and ALP were determined in the serum samples obtained from different groups. The levels of alanine aminotransferase, aspartate aminotransferase, and alkaline phosphatase were significantly elevated in the alcohol administered groups (104.3 ± 17.56 IU/L; 299.45 ± 21.40 IU/L; 259.4 ± 16.40 IU/L, resp.) as compared to the control groups (41.45 ± 15.45 IU/L; 260.34 ± 5.6 IU/L; 155.45 ± 23.40 IU/L, resp.). However, the levels were attenuated in the alcohol treated, free probiotic fed group. More pronounced (*P* < 0.05) results were obtained with AL-CA beads when coadministered during alcohol abuse (31.45 ± 14.59 IU/L; 254.67 ± 38.56 IU/L; 147.65 ± 35.67 IU/L, resp.) (Table 5). The entrapped probiotic appears to cause complete attenuation in the liver markers as no significant difference was observed from the control group.

#### 3.2.4. Tissue Architecture

**Figure 5:**
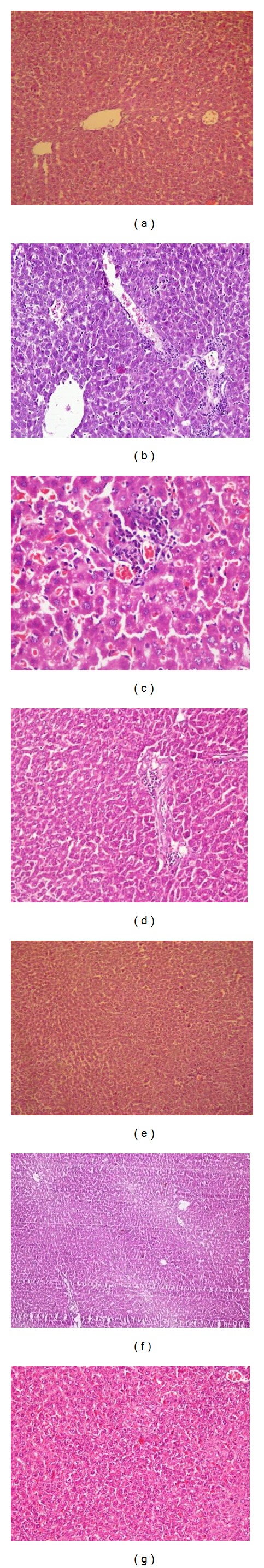
Representative photomicrographs of hematoxylin-eosin stained rat liver sections. (a) Normal rat liver (100x); ((b), (c)) liver section from rat administered 10–14 g/kg of body weight of 35% alcohol orally for 12 weeks showing vacuolar degeneration, microvesicular fatty change, focal collection of lymphocytes, and vascular congestion (200x, 400x), respectively; (d) photomicrograph of alcohol administered cosupplemented with free probiotic group showing normal histology with little hyperplasia of Kupffer cells (100x); (e) photomicrograph of alcohol administered cosupplemented with encapsulated probiotic group showing normal histology (100x); (f) photomicrograph of free probiotic* per se* group showing normal histology (100x); (g) encapsulated probiotic* per se* group showing normal histology (100x).

*Liver*. The liver sections of alcohol administered rats showed vacuolar degeneration, micro- and macrofollicular fatty changes, and focal collection of lymphocytes. Portal tract inflammation (portal triaditis) was also observed (Figures 5(b) and 5(c)). No morphological alteration was observed in the probiotics (free (Figure 5(f)) and encapsulated (Figure 5(g))) group and control group (Figure 5(a)). The probiotic administration in both free and encapsulated groups after alcohol administration showed an improvement in the liver histology (Figure 5(d)). Slight kupffer cell hyperplasia was seen in the free probiotic supplemented group (Figure 5(e)).

**Figure 6:**
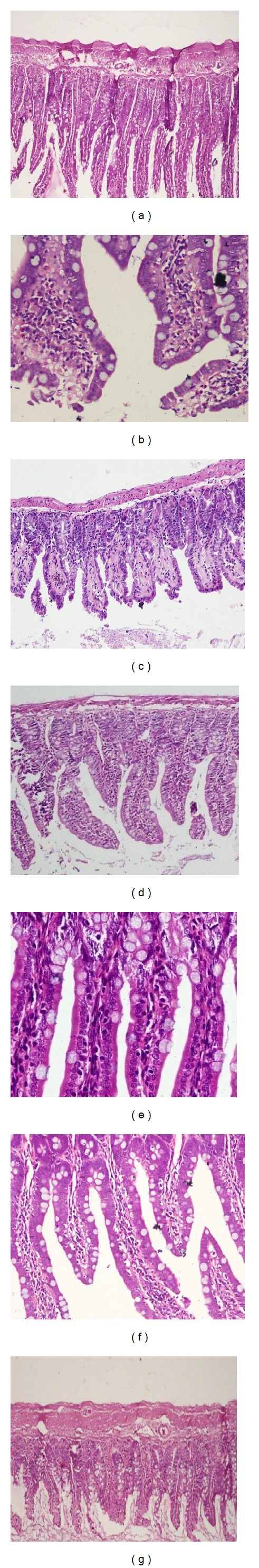
Representative photomicrographs of hematoxylin-eosin stained rat intestine sections. (a) Normal intestine (100x); ((b), (c)) photomicrographs of damaged intestine, severe colitis, and infiltration of lymphocytes in alcohol administered group (400x, 100x); (d) photomicrograph of alcohol administered cosupplemented with free probiotic group showing normal intestine with slight inflammation (200x); (e) photomicrographs of alcohol administered cosupplemented with encapsulated probiotic group showing normal intestine (400x); (f) photomicrograph of free probiotic* per se* group showing normal intestine (200x); (g) photomicrographs of encapsulated probiotic* per se* group showing normal intestine (100x).

*Intestine*. The control group showed normal intestine (Figure 6(a)). The intestine sections of alcohol administered group showed chronic active colitis with excess of lymphocytes especially in the superficial zones of the mucosa. The normal lymphoid follicles seemed enlarged with generally normal glands. However, occasional areas showed necrosis (Figures 6(b) and 6(c)). The intestine of probiotic (free (Figure 6(d)) and encapsulated (Figure 6(e))) treated groups restored the normal intestinal histology with goblet secreting mucous cells. The* per se* group of both the free probiotic supplemented group (Figure 6(f)) and encapsulated probiotic group (Figure 6(g)) showed normal intestine.

#### 3.2.5. Assay for NF-*κ*B

**Figure 7:**
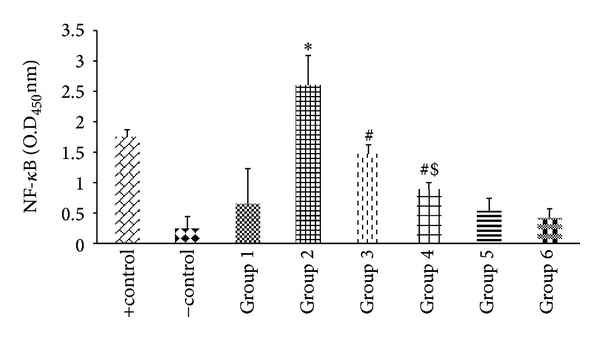
Effect of probiotics on alcohol-induced activation of NF-*κ*B in liver. Values are expressed as mean ± S.D. of five different observations. *P < 0.001 versus group 1, group 5, and group 6; ^#^ *P* < 0.01 versus alcohol (Alc) (group 2); *P* < 0.05 versus group 3. Positive control (+control) refers to the TNF-*α* treated HeLa whole cell extract; negative control (−control) refers to the biotinylated double stranded nonspecific competitor oligonucleotide probe which does not contain the NF-*κ*B consensus sequence. Note: values are not corrected for the protein content. Values are not the same according to the protein content as the value for protein content varying by 14 ± 5.00 mg/mL of tissue for all the samples.

The alcoholic cellular extract showed elevated levels of NF-*κ*B p50 subunit (O.D._450 nm_—2.601) as compared to control group (*P* < 0.001; O.D._450 nm_—0.652), whereas the supplementation of encapsulated probiotic significantly lowered the levels of NF-*κ*B (O.D._450 nm_—0.890). However, the entrapped probiotic decreased the level of NF-*κ*B significantly as compared to the alcohol abused rats and rats coadministered with free probiotic (O.D._450 nm_—1.470) (Figure 7).

#### 3.2.6. Assay for TNF-*α* and IL-12/p40 Subunit

**Figure 8:**
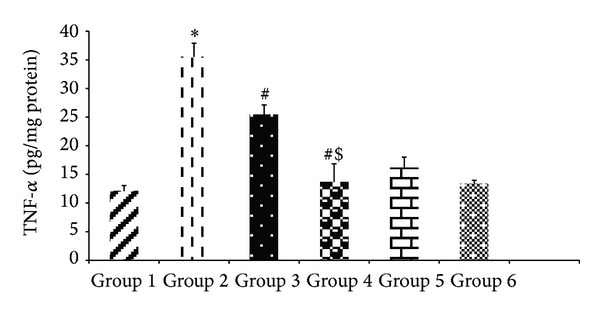
Effect of probiotics on hepatic TNF-*α* levels in alcohol-fed rats. Values are expressed as mean ± S.D. of eight different observations. *P < 0.05 versus group 1, group 5, and group 6; ^#^ *P* < 0.05 versus alcohol (Alc) (group 2); ^$^ *P* < 0.05 versus group 3.

**Figure 9:**
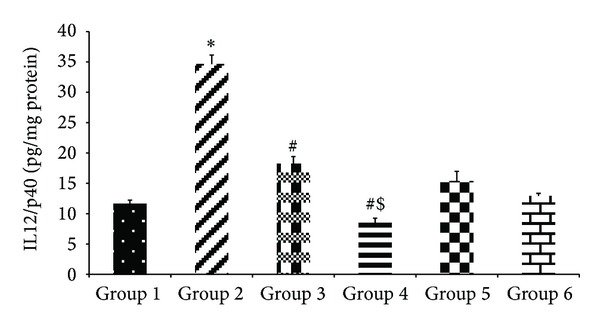
Effect of probiotics on the expression of IL12/p40 on alcohol-induced liver. Values are expressed as mean ± S.D. of eight different observations. *P < 0.05 versus group 1,group 5, and group 6; ^#^ *P* < 0.05 versus alcohol (Alc) (group 2); ^$^ *P* < 0.05 versus group 3.

The levels of both the proinflammatory cytokines, that is, TNF-*α* and IL-12/p40 subunit, decreased significantly (*P* < 0.05) with the cosupplementation of encapsulated probiotic* L. plantarum* as compared to the alcoholic group. Alcohol consumption caused a 2.9-fold rise in the levels of both the cytokines (35.5 ± 2.4 pg/mg protein of TNF-*α* and 34.64 ± 1.5 pg/mg protein IL12/p40) as compared to the control value (12.08 ± 0.98 pg/mg protein TNF-*α* and 11.66 ± 0.56 pg/mg protein IL-12/p40) shown in Figures 8 and 9 but the encapsulated probiotic attenuated the cytokines levels to 13.66 ± 3.2 pg/mg protein of TNF-*α* and 8.46 ± 0.76 pg/mg protein of IL12/p40, respectively. The encapsulated probiotic caused 2.5-fold in the levels of TNF-*α* and 4-fold decrease for IL12/p40 subunit as compared to alcoholic rats.

## 4. Discussion

Bioencapsulation consists of entrapment of a biologically active material inside a microparticle, providing immobilization, safety, and controlled release as well as physical structure or functions. Therefore, in the present study, probiotic was microencapsulated and evaluated for its efficacy in alcohol-induced endotoxin-mediated liver injury.

SEM images revealed that the alginate beads entrapping probiotic have a wrinkled surface. This may be explained by the fact that the particles usually are heterogeneous with a dense surface layer and a loose core which results in their collapse and hence wrinkled shape [22]. The size of the microparticles was found to be in accordance with the earlier study by Lee et al. and Mokarram et al. [22, 23].

AL-CA beads encapsulating the probiotic were found to be more spherical and had a smoother surface than the AL beads. Due to low viscosity, chitosan diffuses rapidly into the microparticles and uniformly distributes itself through the whole shell giving the bead a spherical shape and smoother surface [22]. On coating the alginate beads with chitosan, there was a slight loss of entrapment efficiency which may be due to the removal of superficial probiotic present on the surface of alginate beads during the coating process.

In order to extrapolate the properties of microencapsulated probiotic from* in vitro* to* in vivo* animal models, the beads were tested for their potential to withstand and grow under the simulating conditions encountered in stomach (extreme acidic pH) and intestine (microaerophilic conditions, bile salts). Hence free and encapsulated* L. plantarum* beads were exposed to these conditions. The bacterial population was maintained when encapsulated with alginate and further coated with chitosan, whereas a significant reduction in the count of free probiotic was observed. The free probiotic could not resist the harsh extreme dual environment of the gut which is in accordance with the earlier study [19]. The results are in concordance with Lee et al., 2004 [22], which states that the gastric juice enters the less protected microparticles resulting in a decline in bacterial growth. This indicates that the chitosan coating protected the bacterium from the harsh acidic environment and thus the bacterial population was maintained. Likewise, AL-CA beads have the potential to absorb bile by an ion exchange reaction that takes place between chitosan and bile salts, thus limiting the diffusion of bile salts into the beads and protecting the entrapped probiotic from interacting with the bile salts [24]. Krasaekoopt et al. and Chávarri et al. [19, 25] also reported that the microencapsulation technique provides protection to the probiotic in the harsh gut environments. Chitosan coating did not affect the release of bacteria from within the microparticles. Therefore, it may be concluded that the AL-CA beads provided better protection, stability, and survivability to the probiotic without affecting its release. Thus, these beads were used for subsequent* in vivo* studies.

In the present study, the observed BAL in the rats confirmed the appropriate alcohol consumption which is broken down in the liver generating potentially dangerous by-products in the presence of alcohol dehydrogenase. Perhaps more so than alcohol itself, these products contribute to alcohol-induced liver damage. Moreover, chronic alcohol consumption mediates endotoxemia which occurs due to alterations in the gut microbiota (dysbiosis) as well as compromised gut barrier function leading to increased intestinal permeability. This indicates that the therapeutic strategies targeting the gut microbiome may be effective in the treatment of ALD [26].

In this context, probiotics are being explored extensively in view of their potential to maintain the composition of normal bowel flora, in addition to their competition for nutrient and adhesion sites, production of inhibitory compounds such as bacteriocins, and lowering of cationic pH by the production of short chain fatty acids [27]. It has been documented that the mucoadhesive microparticles such as alginate or chitosan adhere to the intestinal walls thereby increasing the time of absorption. Chitosan, a polycationic polymer, is known to modulate tight junctions controlling the transport process. These factors ensure better bioavailability and sustainability of microparticles inside the gut [28].

In this study, the probiotic beads, because of their nature of controlled release as mentioned above, were found to be better in restoring liver and intestine histology, reducing endotoxemia, and attenuating inflammation. The entrapped probiotic significantly lowered the levels of transcription factor NF-*κ*B after alcohol consumption as compared to the free probiotic which might have reduced TNF-*α* levels. Blocking of NF-*κ*B resulting in the downregulation of TNF-*α* has been reported by us earlier [21]. The results are in accordance with an earlier study where* Lactobacillus delbrueckii* and* Lactobacillus fermentum* ameliorated the inflammation by decreasing concentration of IL-6 and expression of TNF-*α* and NF-*κ*B p65 in ulcerative colitis [29, 30]. VSL#3, a combination of five probiotics, also lowered the expressions of iNOS, COX-2, NF-*κ*B, TNF-*α*, IL-6, and p-Akt and increased IL-10 expression in colonic tissues in acute colitis [31].

The use of antitumor necrosis factor (TNF) therapies has been a huge success in immune-mediated inflammatory diseases (IMIDs) [32]. However, the major limitation with the anti-TNF *α* therapy is that it lacked the efficacy and loss of response in some patients and led to potential adverse effects [33]. Thus, recent attention turned to other significant cytokines released during inflammatory response which are safer and prove to be a pharmaceutical basis for therapeutic intervention. One of the most potent cytokines turns out to be interleukin- (IL-) 12 family of cytokines in the pathogenesis of inflammatory mediated diseases. Interleukin-12 is composed of subunit IL-12 p40, which interacts with the IL-12Rb1 receptor. The cytokines IL-12 and IL-23 share the same subunit IL-12/p40. Blockade of this subunit has been reported to supress both the cytokines [34]. The encapsulated probiotic efficiently reduced the levels of IL-12/p40 subunit which is recently being considered as a potential target for developing novel strategies against ALD. Thus anti-NF-*κ*B, anti-TNF-*α*, and anti-IL-12/p40 subunit activity of the probiotic correlated well with the functional activity of transaminases resulting in restoration of clinical manifestations of the disease in terms of tissue architecture.

## 5. Conclusions

To the best of our knowledge, this is the first report wherein the improved efficacy of probiotic after being microencapsulated in liver damage has been demonstrated. Further, it may be noticed that microencapsulated probiotic ameliorated ALD by suppressing molecular inflammatory markers particularly IL-12/p40 subunit which remained unexplored earlier.

## Figures and Tables

**Table 1:** Size of *L*. *plantarum* alginate beads (AL) and chitosan coated alginate beads (AL-CA).

Bead type	Size of bead in *μ*m
Alginate beads	69.2 ± 6.9 *μ*m
Chitosan coated alginate beads	80.4 ± 1.5 *μ*m*

All values are represented as mean ± standard deviation. *P < 0.05 versus AL beads.

**Table 2:** The drug entrapment efficiency (DEE) of probiotic AL and AL-CA beads (*n* = 6).

S. NO	Alginate conc.	Initial no of bacteria loaded (cfu/100 mg)	No of bacteria entrapped (cfu/100 mg)	EE%
1	1%	10.73 ± 0.17	6.28 ± 0.44	58.5%
2	2%	10.20 ± 0.11	8.15 ± 0.20	80.00%*
3	3%	10.66 ± 0.19	6.61 ± 0.31	62.00%

All values are represented as mean ± standard deviation. *P < 0.01 versus 1%, 3%.

*2% sodium alginate was chosen for entrapment as it maximum entrapment efficiency was obtained. The beads were further coated with chitosan and now the entrapment efficiency obtained was 77%.

**Table 3:** Growth of microencapsulated *L*. *plantarum * (log_10_ cfu) in Simulated Gastric fluid (SGF) and Simulated Intestinal fluid (SIF) (*n* = 6).

	Simulated gastric fluid				Simulated intestinal fluid		
Time	0 hr	1 hr	2 hr	4 hr	5 hr	6 hr	%
Unencapsulated probiotic	8.76 ± 0.11	8.06 ± 0.14	7.79 ± 0.11	7.38 ± 0.2	6.73 ± 0.22	4.25 ± 0.31	56.8%
AL beads	8.59 ± 0.39	8.14 ± 0.23	7.5 ± 0.43	7.2 ± 0.38	6.26 ± 0.34	5.97 ± 0.09^a^	69.4%
AL-CA beads	8.81 ± 0.12	8.4 ± 0.37	8.02 ± 0.16	7.52 ± 0.42	6.41 ± 0.41	6.29 ± 0.13^b,c^	71%

Beads at the end of 4 hours were shifted to SIF. All values are represented as mean ± standard deviation. ^a^ *P* < 0.05 versus unencapsulated probiotic; ^b^ *P* < 0.05 versus AL beads; ^c^ *P* < 0.05 versus AL beads.

**Table 4:** Bile salt tolerance of *L*. *plantarum *(log_10_ cfu) in alginate (AL) and alginate coated chitosan beads (AL-CA) (*n* = 6).

	Initial count	1 hr	2 hr	3 hr	4 hr
Unencapsulated probiotic	8.92 ± 1.23	7.97 ± 0.5	6.91 ± 0.89	6.85 ± 1.54	6.57 ± 0.77
AL beads	8.74 ± 0.37	8.25 ± 0.18	7.85 ± 0.54	7.74 ± 0.16	7.55 ± 0.67^a^
AL-CA beads	8.88 ± 0.65	8.74 ± 0.45	7.95 ± 0.14	7.62 ± 0.49	7.95 ± 0.87^b,c^

All values are represented as mean ± standard deviation in. ^a^ *P* < 0.05 versus unencapsulated probiotic; ^b^ *P* < 0.05 versus unencapsulated probiotic; ^c^ *P* < 0.05 versus AL beads after 4 hours of incubation in 0.3% bile salts.

**Table 5:** Effect of free probiotic and encapsulated probiotic on hepatic markers in the serum of control and alcohol-administered rats.

	Group 1	Group 2	Group 3	Group 4	Group 5	Group 6
ALT (IU/L)	41.45 ± 15.45	104.3 ± 17.56*	57.67 ± 10.56	31.45 ± 14.59^#^	40.62 ± 26.22^#$^	39.56 ± 22.44
AST (IU/L)	260.34 ± 5.6	299.45 ± 21.40*	278.56 ± 32.45	254.67 ± 38.56^#^	265.98 ± 37.56^#$^	258.98 ± 16.80
ALP (IU/L)	155.45 ± 23.40	259.4 ± 116.40*	185.67 ± 40.21	147.65 ± 35.67^#^	158.343 ± 29.56^#$^	152.34 ± 22.45

All values are represented as mean ± standard deviation of eight different observations.

*P < 0.05 versus group 1, group 5, group 6.

^#^ *P* < 0.05 versus group 2.

^$^ *P* < 0.05 versus group 3.
